# A review of supervised machine learning applied to ageing research

**DOI:** 10.1007/s10522-017-9683-y

**Published:** 2017-03-06

**Authors:** Fabio Fabris, João Pedro de Magalhães, Alex A. Freitas

**Affiliations:** 1 School of Computing, University of Kent, Canterbury, Kent CT2 7NF UK; 2 Integrative Genomics of Ageing Group, Institute of Ageing and Chronic Disease, University of Liverpool, Liverpool, L7 8TX UK

**Keywords:** Supervised machine learning, Ageing, Model interpretation

## Abstract

Broadly speaking, supervised machine learning is the computational task of learning correlations between variables in annotated data (the *training set*), and using this information to create a predictive model capable of inferring annotations for new data, whose annotations are not known. Ageing is a complex process that affects nearly all animal species. This process can be studied at several levels of abstraction, in different organisms and with different objectives in mind. Not surprisingly, the diversity of the supervised machine learning algorithms applied to answer biological questions reflects the complexities of the underlying ageing processes being studied. Many works using supervised machine learning to study the ageing process have been recently published, so it is timely to review these works, to discuss their main findings and weaknesses. In summary, the main findings of the reviewed papers are: the link between specific types of DNA repair and ageing; ageing-related proteins tend to be highly connected and seem to play a central role in molecular pathways; ageing/longevity is linked with autophagy and apoptosis, nutrient receptor genes, and copper and iron ion transport. Additionally, several biomarkers of ageing were found by machine learning. Despite some interesting machine learning results, we also identified a weakness of current works on this topic: only one of the reviewed papers has corroborated the computational results of machine learning algorithms through wet-lab experiments. In conclusion, supervised machine learning has contributed to advance our knowledge and has provided novel insights on ageing, yet future work should have a greater emphasis in validating the predictions.

## Introduction

Understanding the ageing process is a very challenging problem in the fields of biology and bioinformatics. Nowadays, with an ever-increasing amount of biological data coming from different high-throughput experiments, it is essential to study this data using machine learning methods that can potentially discover new patterns (or knowledge) in the data, reaching meaningful biological conclusions.

One of the ways machine learning tools can be used to assist biologists understanding the ageing process is through the use of *supervised machine learning algorithms*, which perform classification or regression tasks, as explained in the "Background on supervised machine learning" section. These algorithms use pre-annotated data, for instance, proteins with known functions, to infer the annotations of new, uncharacterized proteins.

In supervised machine learning, the annotated data is called the *training set*, while the unannotated data is the *testing set*. When the annotations are discrete and unordered, they are called *class labels*, when they are continuous numerical values they are called *continuous target (or output) variables*. The training and testing sets comprise *instances*, which in our context are usually proteins or genes. The instances are usually represented by a fixed-size set of numerical or nominal variables, each variable in this set is called a *feature (or predictor)*, and represents a property of an instance. For example, it is common to represent proteins (the instances) using as features physicochemical properties of their amino acid sequence (the features) and as annotations Gene Ontology terms (the class labels) associated with the instances.

In summary, supervised machine learning algorithms use the features and annotations in the training set to induce a model to predict the annotations of the instances in the testing set.

Besides being useful for inference, supervised machine learning algorithms may have the additional purpose of discovering interpretable knowledge. For instance, experts can interpret the results of such algorithms to find patterns to classify a protein as ageing-related, or to investigate the relative importance of features used to predict the chronological age of individuals.

In this paper we review works that use supervised machine learning to study ageing-related proteins and, at the same time, interpret some part of the supervised machine learning results in order to gain biological insights to help understanding the very complex ageing process.

Machine learning experiments are relatively fast; and they can make predictions that help to suggest promising wet-lab experiments to be done. This approach is cost effective, since wet-lab experiments are in general much slower and expensive than computational experiments. Furthermore, we argue that a stronger integration between machine learning experts and biologists to corroborate the prediction of machine learning algorithms is necessary to validate the current practice in the field.

We organise this paper as follows: in the "Background on supervised machine learning" section we give some background knowledge on supervised machine learning. The "Supervised machine learning applied to ageing research" section presents the types of supervised machine learning problems we have identified in our review. The "Biological insights derived from supervised machine learning algorithms" section reviews the main biological conclusions reported in the papers we have analysed. In "Discussion and conclusions" section we summarise our findings and draw our final conclusions.

## Background on supervised machine learning

**Fig. 1:**
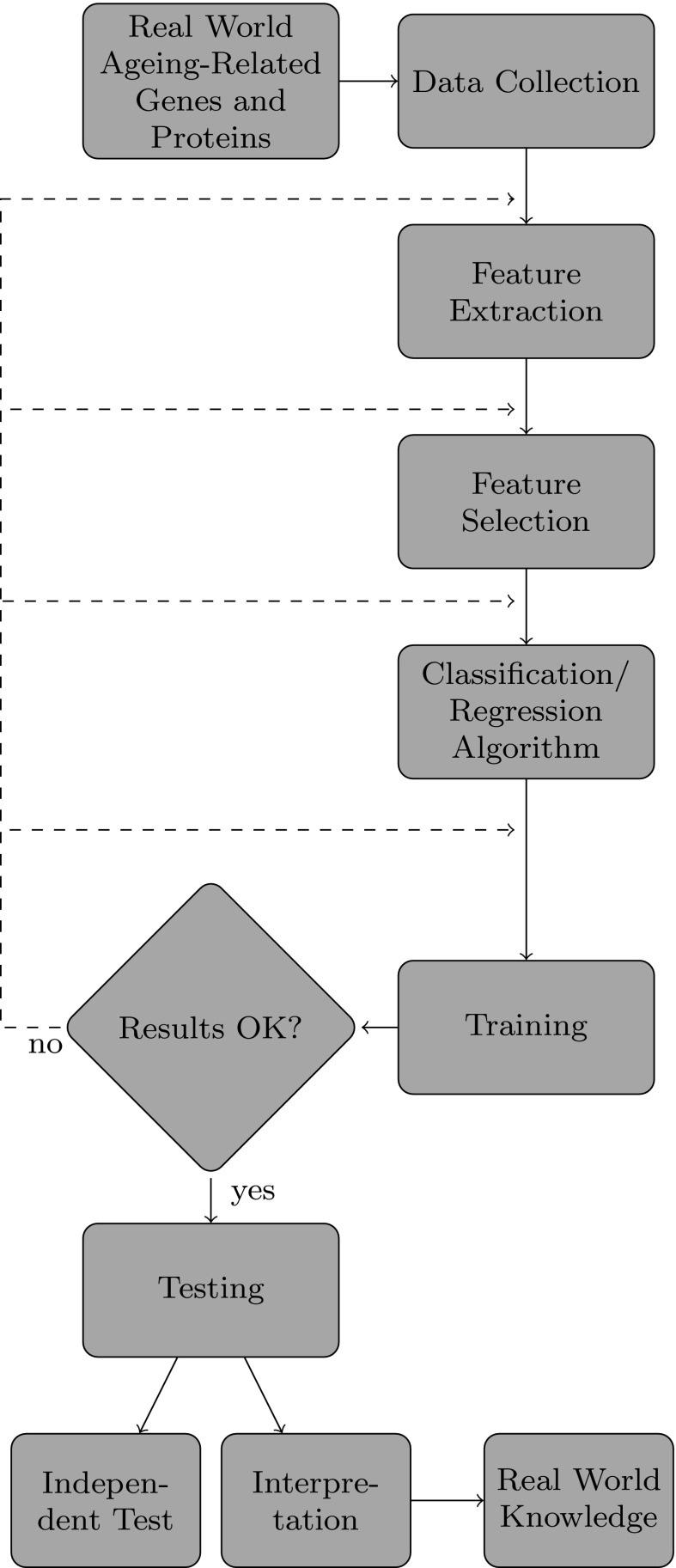
Overview of the supervised learning process, adapted from (Kuncheva 2004)

When dealing with problems with significant amounts of data, like studying ageing-related genes/proteins, it is often desirable to have some type of automated, principled, data-driven way of discovering knowledge that assists the user reaching meaningful biological conclusions. *Supervised machine learning* algorithms can be used to this end.

Supervised machine learning consists of methods for automatically building a predictive function $\mathcal {F: X \rightarrow Y}$ that maps $\mathcal {X}$ (the *predictor attributes* of an instance), to a prediction $\mathcal {Y}$ (the *target variable* of an instance), given a set of training instances (*the training set*) represented by tuples $(\mathcal {X}_i, \mathcal {Y}_i)$, where $\mathcal {Y}_i$ is the target variable and $\mathcal {X}_i$ is the vector (typically containing numerical and/or categorical values) encoding the predictor attributes (features) associated with the *i*-th instance (Witten et al. 2011).

For instance, if the supervised machine learning task is to predict if a protein (an instance) is ageing related or not (the target variable), one can use a set of proteins that are known to be ageing related or not (instances in the training set), build a model $\mathcal {F}$, and use $\mathcal {F}$ to get the predictions for a set of proteins that were not used during training (the testing set).

The task is called *classification* when the target variable $\mathcal {Y}$ is categorical (nominal or discrete) and called *regression* when $\mathcal {Y}$ is continuous (real-valued). In the works we have reviewed, some authors treat the problem as a *binary* classification task (e.g., Freitas et al. 2011), that is, the variable $\mathcal {Y}$ can take only two possible discrete values. Others deal with *hierarchical* classification problems (e.g., Fabris and Freitas 2016), where $\mathcal {Y}$ takes nominal or discrete values that are organised into a pre-defined hierarchy. Some works treat the problem as a regression task rather than a classification task (e.g., Nakamura and Miyao 2007). There are other types of supervised machine learning problems (Witten et al. 2011), however, we focus on these three, as they were the only ones used in the papers we reviewed.

The pre-processing phase of classification and regression algorithms involves two important steps: first, in the *feature extraction* phase, numerical features are extracted from the unprocessed data. Second, a *feature selection* algorithm is sometimes used. Feature selection algorithms work by using some statistical approach to find correlations between the features (predictor attributes) and the target variables, eliminating features with low predictive power. It is well-known that using feature selection algorithms often (but not always) improves the predictive performance of $\mathcal {F}$, as using redundant and irrelevant features often degrades the predictive performance of $\mathcal {F}$ (Liu and Motoda 2012).

It is expected that the predictive function $\mathcal {F}$ will approximate the real distribution of the target variable, given the values of an instance, by finding correlations between features and the target variable. It is worth mentioning that the predictive performance of the function $\mathcal {F}$ should be estimated by using a test set, a set of labeled instances that was not used to build $\mathcal {F}$. One should trust the conclusions extracted from $\mathcal {F}$ only if $\mathcal {F}$ was proven to be a good predictor of $\mathcal {Y}$ given $\mathcal {X}$ on the test set.

Figure 1 presents the previously discussed workflow graphically. Note that the workflow is iterative. Typically, many iterations are needed, training a classification/regression algorithm(s) with different parameters and possibly different subsets of features in different iterations, until the predictive function $\mathcal {F}$ built from the training set is considered to have satisfactory predictive accuracy.

Once the final function $\mathcal {F}$ has been built and its predictive performance has been estimated on the test set, $\mathcal {F}$ can be further validated on an *independent* test set, for instance using data from different species.

In addition, sometimes the predictive function $\mathcal {F}$, or part of the workflow leading to the construction of $\mathcal {F}$, can be interpreted to extract meaningful biological knowledge. For instance, some predictive functions like decision trees or IF-THEN rules can be directly interpreted by the user (Freitas 2013; Fabris et al. 2016). The feature selection process leading to the construction of the predictive function can also be exploited to analyse which features are more important to model the problem at hand, being a good starting point for understanding the underlying biological processes being modeled by the predictive function. Note that the validation of $\mathcal {F}$ on an independent test set and the biological interpretation of $\mathcal {F}$ are often missing in the literature.

Achieving perfect predictive accuracy performance is rare in machine learning problems. For this reason, it is important to always use some kind of predictive performance measure to assess the quality of $\mathcal {F}$. In fact, it is not uncommon for classification and regression algorithms to have poor performance on the test set (and on the independent test set, if this is used) when the underlying assumptions that such algorithms make are violated. Examples of conditions that may lead to such violations are: existence of uninformative features, bad algorithm parameter choices, incompatibility between algorithm choice and the training data, insufficient training data, as well as training and test sets (or independent test sets) following different statistical distributions—called the “data shift” problem (Quionero-Candela et al. 2009). In addition, supervised learning algorithms have the disadvantage of requiring a relatively large amount of labelled data.

Usually it is not feasible to check if all assumptions made by supervised machine learning algorithms are satisfied before inducing a model using the available training data. For this reason, in practice, it is not possible to know which supervised learning algorithm will be the best one for a dataset. Thus, it is always considered good practice to test more than one supervised machine learning algorithm (ideally with different biases) to pick the model that better fits the data, using some measure of predictive performance.

As commonly used measures of predictive performance for binary classification we can cite accuracy, precision, recall, F-measure, and the Area Under the ROC Curve (AUROC).

*Accuracy* is the number of correct predictions divided by the total number of predictions. This measure is simple to understand but should be avoided when the class distribution is skewed—i.e., one class is much more frequent than the other. In this scenario, models that are better at predicting the minority class (which is usually the class of interest) are over-penalised in comparison with models that are more conservative, rarely predicting the minority class. When the class distribution is unbalanced, one can consider measures based on *precision* and *recall*.

Let the positive class be the class of most interest. *Precision* is the number of correct positive predictions the classifier has made divided by the total number of positive predictions. *Recall* is the number of correct positive predictions divided by the total number of instances with the positive class. Note that a classifier can have the perfect *recall* of 1.0 by simply predicting every instance as the positive class. Likewise, a classifier can have a very high *precision* by being very conservative, only predicting the positive class of ‘easy’ instances. For this reason, it is recommended to use predictive accuracy measures that combine *precision* and *recall*. The *F-measure* combines *precision* (*Pr*) and *recall* (*Re*) by calculating their harmonic mean, as shown in the following formula: $F=\frac{2 \times Pr \times Re}{Pr+Re}$.

Note that the previously defined measures of predictive accuracy only work when considering ‘crisp’ predictions. Other measures, such as the AUROC measure, deal with probabilistic predictions, that is, when every class is predicted with an associated probability. The AUROC measure works by calculating the area under the curve defined by the true positive rate and false positive rate (Witten et al. 2011), when varying a probability threshold that defines whether an instance is predicted to have the positive or negative class.

For regression, among the most common predictive accuracy measures are the mean squared error (MSE) and the adjusted Coefficient of Determination (adjusted $R^2$). These measures assess the level of agreement between the predictions of the regression algorithms and the actual value of the target variable.

The MSE is the mean squared difference between the predicted target values and actual target values. It has the advantage of being easy to understand but the downside of having an unbounded range, being hard to analyse without a reference. The adjusted $R^2$, on the other hand, measures the proportion of data variance the regression model can explain. The range of the adjusted $R^2$ is the interval [0, 1].

For more information about these and other measures of predictive accuracy, please refer to Tan et al. (2006).

## Supervised machine learning applied to ageing research

In this section we present a categorization of papers studying the biology of ageing according to the different types of target variables used in the papers we reviewed.

The inclusion criteria we adopted were the following: First, the paper must have used a supervised machine learning algorithm during the process of studying the biology of ageing. The work might use the supervised machine learning algorithm as the main source of biological insight (e.g., Fabris et al. 2016) or as an essential part of a larger workflow studying the biology of ageing (e.g., Huang et al. 2012). Second, the paper must have discussed at least some part of the predictive model built by the algorithm in the context of the ageing literature. Papers that just report a predictive accuracy measure for the built model(s), without interpreting it (them), are not the focus of this review.

Ageing is a complex biological phenomena: it is the result of multiple interacting genetic and environmental factors. Due to this complexity, ageing has been studied at several levels of abstraction using supervised machine learning algorithms, both in the definition of the types of predictor attributes (features) and in the definition of the target variable.

To define predictor attributes, some works use low-level features derived from “raw” amino-acid sequences of ageing-related proteins (e.g., Fabris and Freitas 2016). Other works use biomarkers of higher-level biological systems like metabolic and renal systems (e.g., Putin et al. 2016). Some authors even use non-traditional hierarchical features to represent instances, exploring the hierarchical relationships among gene functions available in curated ontologies, such as the Gene Ontology (GO) (e.g., Wan and Freitas 2013).

In this work we focus more on the types of target variables used in ageing research. Although the use of interpretable predictor attributes is essential for reaching biological conclusions, this topic has been explored in other works about machine learning applied to general biological research (Pandey et al. 2006), which could be used as a reference for a biologist using machine learning for studying the ageing problem. On the other hand, a categorisation of the type of target variables to study ageing has never been proposed, as far as we know.

**Fig. 2:**
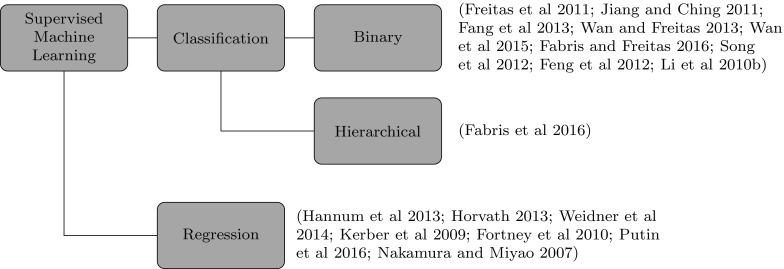
Categorisation of works using supervised machine learning applied to the biology of ageing

**Table 1:** Main data analysis characteristics of papers that focus on applying some supervised machine learning algorithm to tackle a biological ageing problem and then interpret the results to get some type of biological insight about the ageing process

Type of sup. machine learning problem	References	Paper’s title	Supervised learning algorithm	Feature type	Species
Binary classification problem (involving DNA repair and ageing-related proteins)	Freitas et al. (2011)	A data mining approach for classifying DNA repair genes into ageing-related or non-ageing-related	Decision tree (J48)	Protein–protein interactions (PPI), Gene Expression, Gene Ontology terms, type of DNA Repair, Dn/Ds ratio	Human
Binary classification problem (involving DNA repair and ageing-related proteins)	Jiang and Ching (2011)	Classifying DNA repair genes by kernel-based support vector machines	SVM (Support vector machine)	Gene expression levels	Human
Binary classification problem (involving DNA repair and ageing-related proteins)	Fang et al. (2013)	Classifying aging genes into DNA repair or non-DNA repair-related categories	Feature selection based on random forests	Protein-protein interactions (PPI)	Human
Binary classification using hierarchical features (pro-longevity vs. anti-longevity proteins)	Wan and Freitas (2013)	Prediction of the pro-longevity or anti-longevity effect of *Caenorhabditis Elegans* genes based on Bayesian classification methods	Hierarchical feature selection used in the first phase of a naive Bayes algorithm	Gene Ontology terms	Worm
Binary classification using hierarchical features (pro-longevity vs. anti-longevity proteins)	Wan et al. (2015)	Predicting the pro-longevity or anti-longevity effect of model organism genes with new hierarchical feature selection methods	Hierarchical feature selection used in the first phase of a naive Bayes algorithm	Gene Ontology terms	Worm, fly, mouse, yeast
Hierarchical classification (using proteins as instances and ageing-related GO terms as classes)	Fabris et al. (2016)	An extensive empirical comparison of probabilistic hierarchical classifiers in datasets of ageing-related genes	Decision tree for hierarchical classification	Protein-protein interactions	Worm, fly, mouse, human, yeast
Binary classification (ageing-related vs. non-ageing-related mortality-related proteins)	Fabris and Freitas (2016)	New KEGG pathway-based interpretable features for classifying ageing-related mouse proteins	Decision table	KEGG pathway features	Mouse
Binary classification (ageing-related vs. non-ageing-related genes)^a^	Song et al. (2012)	Discovering aging-genes by topological features in *Drosophila melanogaster* protein-protein interaction network	SVM	PPI Network features	Fruit fly
Binary classification (ageing-related vs. non-ageing-related genes)^a^	Feng et al. (2012)	Topological analysis and prediction of aging genes in *Mus musculus*	SVM	PPI Network features	Mouse
Binary classification (ageing-related vs. non-ageing-related genes)^a^	Li et al. (2010b)	Computational prediction of aging genes in human	SVM, k-NN, Decision tree	PPI Network features	Human
Binary classification (Longevity vs. non-longevity genes)	Li et al. (2010a)	Systematic analysis and prediction of longevity genes in *Caenorhabditis elegans*	SVM, k-NN, Decision tree	Functional interaction network features, conservation score	Worm
Two-layer binary classification (life span change and then increase or decrease the life span of genes)	Huang et al. (2012)	Deciphering the effects of gene deletion on yeast longevity using network and machine learning approaches	Selected features using k-NN with Incremental feature selection	PPI Network, biochemical, physicochemical, functional, and deletion features	Yeast
Regression (prediction of rate of ageing)	Nakamura and Miyao (2007)	A method for identifying biomarkers of aging and constructing an index of biological age in humans	Logistic regression	Various physiological biomarkers	Human
Regression (prediction of chronological age)	Hannum et al. (2013)	Genome-wide Methylation Profiles Reveal Quantitative Views of Human Aging Rates	Elastic net	Methylome profile	Human
Regression (prediction of chronological age)	Horvath (2013)	DNA methylation age of human tissues and cell types	Elastic net	Methylome profile	Human
Regression (prediction of chronological age)	Weidner et al. (2014)	Aging of blood can be tracked by DNA methylation changes at just three CpG sites	Unspecified regression algorithm	Methylome profile	Human
Regression (prediction of chronological age)	Fortney et al. (2010)	Inferring the functions of longevity genes with modular subnetwork biomarkers of *Caenorhabditis elegans* aging	SVR (support vector regression)	Modular features from gene interaction networks	Worm
Regression (prediction of chronological age)	Putin et al. (2016)	Deep biomarkers of human aging: Application of deep neural networks to biomarker development	Deep neural network	Features extracted from standard blood tests	Human
Regression (prediction of chronological age and survival)	Kerber et al. (2009)	Gene expression profiles associated with aging and mortality in humans	LASSO regression algorithm	Gene expression profiles	Human

Columns 1 and 4 to 6 inform us, respectively, the type of supervised learning problem that was considered in the paper, the supervised learning algorithm whose results were interpreted, the feature type used by the algorithm and the species that were considered in the interpretation

Note that a paper may contain other machine learning algorithms, feature types and species whose results were not interpreted and therefore are not listed in the table

^a^These works also include analysis of individual features

Figure 2 shows the full characterization of the works we considered in the three types of supervised machine learning task (binary classification, hierarchical classification and regression) we are studying. Table 1 contains the full list of works being considered in this paper with supplementary information about each work. In the next sections we go into detail on each type of target variable present in the works we reviewed.

### Binary classification

The majority of works we reviewed uses a binary classification algorithm. Arguably, using binary target variables facilitates interpretation as the user does not have to deal with the complexities of a larger number of class labels (sometimes hundreds) when interpreting the model. For instance, in the hierarchical classification problem studied in (Fabris et al. 2016), several hierarchical classes are predicted at the same time, with different probabilities, so extras steps are required to select which classes to focus the interpretation on. It can be argued, however, that some information is lost when not using a larger number of class labels or hierarchical classes (see "Hierarchical classification" section).

When using binary classification, the first task is to define the classes you wish to predict/distinguish between. Next we list how authors have defined these classes in the works we reviewed.

#### Ageing-related DNA repair

Some works (Freitas et al. 2011; Jiang and Ching 2011) have built classification models to allow the discrimination of ageing-related and non-ageing-related DNA repair genes. In these works, the positive class is defined as DNA repair genes that are also related to ageing, while the negative class comprises DNA repair genes that are not related to ageing. This differentiation is important because understanding why some DNA repair genes are ageing-related, while others are not, can help biologists pinpoint the molecular causes or mechanisms of ageing and some progeroid syndromes (accelerated ageing).

In Fang et al. (2013) the authors propose a different but related discrimination, classifying known ageing genes into DNA repair or non-DNA repair related. In other words, the negative class is “ageing-related non-DNA repair”, instead of “non-ageing related DNA repair”; while the positive class is the same as in Freitas et al. (2011), Jiang and Ching (2011).

#### Pro-longevity proteins

Other works (Wan and Freitas 2013; Wan et al. 2015) consider pro-longevity vs. anti-longevity class labels when constructing the binary-class datasets. Pro-longevity genes are defined as the genes whose over-expression extends lifespan, or whose decreased activity reduces lifespan. Anti-longevity genes have the opposite effects Tacutu et al. (2013). This definition of positive and negative instances is interesting to uncover properties that define proteins as pro-longevity or anti-longevity. However, a predictive model built for this binary classification naturally has the weakness that it is not suitable for classifying all proteins of an organism: as the majority of proteins are not pro- nor anti-longevity, models trained without these proteins would likely return incorrect predictions for many proteins with unknown longevity effect.

To address this problem, in Huang et al. (2012) the authors introduce an additional classifier prior to passing instances to the pro-/anti-longevity classifier. This layer differentiates between lifespan change and no lifespan change. This extra layer complicates model interpretation but enables the use of the classification model in datasets with a larger and more diverse set of proteins.

Another type of target variable definition we have encountered (Li et al. 2010a) considers as positive “pro-longevity” proteins and as negative proteins that are not “pro-longevity”, regardless of whether or not they have an “anti-longevity” effect.

#### Ageing-related proteins

In Fabris and Freitas (2016) the authors consider as positive instances proteins that are involved in increased mortality and ageing, and as negative instances proteins that are involved in mortality and *not* involved in ageing. It is interesting to study what differentiates these two classes, since some mutations reduce the lifespan of organisms (e.g., they increase the incidence or lethality of some diseases) but are believed not to be related to ageing.

### Hierarchical classification

Typical classification problems involve a flat set of class labels, i.e., there is no hierarchical relationships among the class labels to be predicted. By contrast, in hierarchical classification problems, the set of class labels is organized into a hierarchy, usually a tree or a DAG (Directed Acyclic Graph), where each node represents a class label and the edges represent generalization-specialization relationships among classes. Dealing with hierarchical classes is common when studying the ageing process, since the main ontology used to annotate ageing-related proteins is the GO, which is organized as a DAG where, broadly speaking, nodes represent functions or processes performed by genes or proteins, and edges represent specialisation-generalisation relations between those functions or processes.

Usually authors tend to ignore the hierarchical organisation of the GO and deal only with flat classes, which are easier to interpret and to work with, as traditional classification algorithms can be used. However, hierarchical classification algorithms that exploit hierarchical class relationships can achieve higher predictive accuracies than “flat” classification algorithms (Silla Jr and Freitas 2011).

Hierarchical classification algorithms may be divided into two broad types (Silla Jr and Freitas 2011): global or local. Local hierarchical classification (LHC) algorithms first build a set of local classification models (base classifiers) by training a traditional (flat) classification algorithm for each (typically small) part of the class hierarchy in the training phase. Then they combine all the local predictions during the testing phase, when predicting the class of a new instance. By contrast, global hierarchical classification algorithms build a single global classification model predicting classes in the whole class hierarchy.

Global hierarchical classification algorithms have the advantage of producing a single coherent global classification model, which tends to be more easily interpreted than a large number of different local classification models.

The work of Fabris et al. (2016), the only one dealing with hierarchical classification of ageing-related genes/proteins and interpreting the corresponding classification model, uses a global hierarchical decision tree model to classify ageing-related proteins in hierarchical classes. The classes are ageing-related because they are the over-expressed hierarchical classes present in the ageing-related proteins from the GenAge database (de Magalhães et al. 2009a).

### Regression

Recall that in regression problems the target variable is continuous (real-valued), whilst in classification problems the target variable is categorical (nominal or discrete).

In ageing research, regression techniques have been used to predict chronological age given a set of biomarkers (Fortney et al. 2010; Putin et al. 2016; Weidner et al. 2014; Horvath 2013; Kerber et al. 2009; Hannum et al. 2013) and to build an index of the rate of ageing given a set of biomarkers (Nakamura and Miyao 2007).

It is important to identify which biomarkers are most related to ageing phenotypes, thus enabling, for instance, the use of biomarkers to measure the results of interventions in ageing-research and to possibly identify genes that are related to ageing when over- or under-expressed.

In addition, some biomarkers (such as microarray gene expression profiles) can be used in regression algorithms to identify particular functions and processes that have modified activity with age. Examples are increased inflammation and immune responses and decreased energy metabolism with age (de Magalhães et al. 2009b). This works by first identifying the set of genes with altered expression with age and next associating this set of genes with a particular ageing process or change. An example of this approach can be seen in Kerber et al. (2009).

## Biological insights derived from supervised machine learning algorithms

### Supervised machine learning findings support the link between ageing/longevity and specific types of DNA repair

The link between DNA repair and ageing/longevity is well established in the biological literature: it has been shown that some ageing-related diseases in humans are directly linked to malfunctioning pathways related to DNA maintenance—e.g. some progeroid (accelerated ageing) syndromes are caused by mutations in DNA-repair-related genes (Lombard et al. 2005; Freitas and de Magalhães 2011). Moreover, it has been shown that over-expression of DNA-repair-related genes increase lifespan in some animal species and that DNA-repair efficiency is positively correlated with increased longevity in several species (Shaposhnikov et al. 2015).

In Freitas et al. (2011), the authors noted, after analysing the model generated by the decision tree algorithm J48 (induced to differentiate between ageing-related DNA repair genes and non-ageing-related DNA repair genes in humans), that if a DNA repair gene’s protein product interacts with *XRCC5* (Ku80), that gene is likely ageing-related.

Interestingly, links between Ku proteins and longevity have been found by other supervised machine learning works studying connections between DNA repair genes and ageing in humans. In Jiang and Ching (2011), the authors use an SVM algorithm to distinguish between human ageing-related DNA repair genes and human non-ageing-related DNA repair genes. By analysing the instances (proteins) furthest from the SVM’s hyperplane, the authors identified that *XRCC6* (Ku70) and *MLH1* are strongly predicted as ageing-related. Ku70, Ku80 (from Freitas et al. 2011) and *MLH1* are involved in non-homologous end joining (Bannister et al. 2004; Fattah et al. 2010). Interestingly, the Ku protein family is highly conserved among eukaryote species and is a well-conserved longevity regulator across species, being a key target of ageing research (Dynan and Yoo 1998). The authors also point out that *PARP1*, *PCNA* and *APEX1* are essential to base excision repair, a pathway that is known to be affected by deficient WRN proteins, which are directly linked to Werner syndrome, a disease characterized in humans by accelerated ageing.

Not surprisingly, a homolog of *WRN* (*WRN1*) has been identified as longevity-related in the worm while classifying worm genes into longevity-related and non-longevity-related in Li et al. (2010a). Interestingly, however, defects in the WRN protein in mice do not cause, by themselves, Werner-like phenotypes. However, in conjunction with defects in p53 they do cause the typical Werner syndrome phenotypes (Lombard et al. 2000). This stresses the already known fact that even in relatively closely related species (like mouse and human) ageing-related genes in one species may not directly lead to the same ageing phenotype in another de Magalhães (2014).

Still regarding WRN and p53, in (Fabris et al. 2016) the authors noted (by interpreting a classification model) that interaction with p53 is a good predictor of ageing-related GO terms in humans and mouse. In both human and mouse, p53 is closely related to WRN, and both proteins participate in DNA repair, reinforcing the importance of WRN to predict ageing-related GO terms in humans.

Interestingly, two works analysing the methylation profile of human subjects have identified that methylation sites in close proximity to genes associated with DNA repair are good predictors of chronological age. This suggests that changes in the expression levels of these genes are closely associated with ageing in humans (Horvath 2013; Hannum et al. 2013).

In Kerber et al. (2009), the authors highlight that the expression of the genes *TERF2IP*, *CBX5*, *AURKB* and *CDC42*, which are all related to genomic maintenance in humans, are important to predict chronological age.

In Wan et al. (2015) one of the features selected to classify proteins as ageing-related in yeast was the GO term “double-strand break repair”, directly related to DNA-repair.

In Huang et al. (2012), “mitochondria genome maintenance”, also related to DNA maintenance, was one of the features selected to predict the effect of a gene deletion as a “change” or “no change” of lifespan in yeast.

In Fang et al. (2013), the authors used the Gini index calculated by the Random Forest algorithm to select the 18 most relevant Protein-Protein-Interaction (PPI) features. Out of these 18 features, the authors highlighted interactions with proteins BLM, ERCC2, FANCG, MSH2, ATM, MRE11A and ATR, which play a role in check-point control and DNA damage check; and interactions with proteins BLM, WRN, MRE11A and Mre11, which are associated with the maintenance of telomeres (Fang et al. 2013).

In summary, it appears that the results of supervised machine learning algorithms have corroborated the fact that DNA repair is strongly linked to ageing/longevity. DNA repair-related features are commonly chosen as good predictors of ageing/longevity by classification algorithms. Furthermore, proteins related to DNA-repair and maintenance are commonly predicted as ageing-related. To some extent, the machine learning algorithms are reflecting a bias stemming from the biological knowledge already encoded in the data. However, the algorithms can also find proteins highly related with DNA repair that are not annotated as ageing-related [like the ones studied in Li et al. (2010a)]. In fact, in Li et al. (2010a), the authors proceeded to carry out wet-lab experimentation of proteins predicted to be longevity-related in worms, identifying two proteins that increase lifespan in the animal, namely: VPS-34 and PHI-38. In addition, the analysis of the classification models built by the algorithms suggests that, out of the different types of DNA repairs, non-homologous end joining seems to be the one most relevant for the ageing process.

### Ageing-related proteins tend to be highly connected and are enriched for certain functions

Other important type of biological conclusion derived by supervised machine learning algorithms is how ageing-related proteins are connected both between themselves and with non-ageing related proteins.

In Li et al. (2010a), the authors conclude (with statistical support) that several proteins’ properties derived from interaction graphs (topological features) and sequence analysis have different distributions, depending on whether or not they are longevity-related. Next we summarize the main biological conclusions the authors have drawn for longevity-related proteins or genes in worms:

(1) Longevity-related proteins (LRPs) have, on average, more interaction partners than non-LRPs. (2) LRPs have more LRPs in their neighborhood than non-LRPs. (3) LRPs tend to be closer between themselves than to other proteins. (4) LRPs tend to occupy a more central location in the PPI graph. (5) LRPs tend to be expressed together. (6) LRPs tend to have longer amino acid sequences. (7) Longevity-related genes tend to be more conserved. (8) Longevity-related genes are enriched for certain functions (e.g. Negative regulation of cell proliferation, Positive regulation of non-apoptotic programmed cell death, Cilium biogenesis and regulation). (9) Longevity-related genes tend to show certain RNAi phenotypes (e.g. abnormal DAF dauer formation, abnormal transgene expression, and variable embryonic terminal arrest).

Similarly to Li et al. (2010a), in Song et al. (2012), Feng et al. (2012), Li et al. (2010b), the authors use topological network features to study the ageing process. The latter works, however, predict ageing-related genes vs. non-ageing-related genes, not longevity vs. non-longevity genes. The difference between ageing-related genes and longevity-related genes is subtle but important: an increase in longevity may not be a result of changes in the ageing process. For instance, longevity may be increased by mutations that improve resistance against some disease, not altering the overall ageing process (de Magalhães et al. 2009a). Also, the works in Song et al. (2012), Feng et al. (2012), Li et al. (2010b), study fruit flies, mice and humans, while (Li et al. 2010a) studies worms. Next we summarize the biological conclusions drawn in Song et al. (2012), Feng et al. (2012), Li et al. (2010b), all with statistical support.

(1) For fly, mouse, and human; ageing-related proteins (ARPs) have, on average, more interaction partners than non-ageing-related proteins. (2) For fly and mouse; ARPs tend to be in more tightly connected protein clusters than non-ARPs. (3) For fly, mouse, and humans; ARPs have more ARPs in their neighborhood than non-ARPs. (4) For fly; ARPs tend to be closer between themselves than to other proteins. (5) For fly and human; ARPs tend to occupy a more central location in the PPI graph. (6) For fly and mouse; ARPs behave more like hubs than non-ageing related proteins. (7) For human; ARPs tend to be expressed together. Hence, despite the differences in species and data, the conclusions in Song et al. (2012), Feng et al. (2012), Li et al. (2010b), are broadly similar to the conclusions in Li et al. (2010a).

In Fortney et al. (2010), the authors show that their technique of creating modular subnetworks of longevity genes creates modules with statistically significantly more longevity genes. Also, they claim, with statistical support, that modular subnetworks participate in many different age-related biological processes.

In Huang et al. (2012), the authors indicate that the edge density and edge weight density of the deletion network and the local centrality of a deletion gene can be used to predict ageing-related effects of gene deletion.

In summary, all these works point to the fact that ageing-related proteins tend to be more connected than other proteins and also appear to be closer to each other in protein-protein interaction networks than other proteins.

### Autophagy and apoptosis mechanisms are associated with ageing and longevity

The mechanisms of autophagy and apoptosis control the turnover of organelles and programmed cell death, respectively. Several works have identified that autophagy/apoptosis-related proteins are related to ageing (Kurz et al. 2007). Next we review the main biological conclusions regarding these processes drawn from supervised machine learning works.

In Li et al. (2010b), the authors classify human proteins as ageing-related or non-ageing-related using SVMs. They highlight that protein “VPS-34”, involved in autophagy (Jaber and Zong 2013), was predicted as ageing-related with a probability of 0.93.

In Feng et al. (2012), where the authors classify mouse proteins as ageing-related or non-ageing-related using SVMs, they mention that the gene *Akt1* was predicted as ageing-related with high probability, and this gene is strongly involved in apoptosis (Xu et al. 2002).

In Wan and Freitas (2013), among the top ranking terms selected to predict the pro-longevity effect of a gene in worms were “autophagy” and “apoptotic process”, once again reinforcing the link between ageing and autophagy/ apoptosis. In Wan et al. (2015), the authors identified “apoptotic signaling pathway” as a good anti-longevity predictor for worms.

In Fang et al. (2013), interactions with a number of proteins, including BLM, ATM, RPA1, PCNA and HSPA4, which are linked to the apoptosis of tumor cell lines, were found to be good predictors of DNA-repair ageing-related proteins.

In Fabris and Freitas (2016), for mouse, it was found that if a protein has any influence on CDK1 (associated with apoptosis), then that protein is more likely to be ageing-related. CDK1 is putatively associated to ageing in mice (Xiao et al. 1999).

In Kerber et al. (2009), the authors highlight that expression of the genes *CORO1A* and *AURKB* (associated with apoptosis) are good predictors of chronological age.

In summary, several works have identified that proteins related to apoptosis and autophagy are good predictors of ageing-relatedness. This is in agreement with the literature, as these mechanisms are essential to tumor suppression and regulation of oxidative stress (Filomeni et al. 2015), which in turn, are linked to the ageing process.

### Interactions with nutrient receptor genes are good predictors of ageing-relatedness

The link between nutrient sensing and ageing is well-established in several organisms: mutations in genes responsible for nutrient signalling and food recognition promote longevity across species López-Otín et al. (2016). The following works identified protein features (functional annotations) related to nutrient signalling that are good predictors of a role in the ageing process.

In Fabris et al. (2016), the authors identify that in fruit flies some over-represented GO terms are involved in food recognition. The work in Wan et al. (2015) shows that in fruit flies, “sensory perception”, which is essential to food recognition, was among the top selected features using their feature selection algorithm.

The work in Wan and Freitas (2013), studying worms, shows that one of the top features selected by their feature selection algorithm is “response to nutrient level”.

In summary, not surprisingly, proteins involved in nutrient sensing have been found to be good predictors of ageing-relatedness, since the connection between nutrient sensing and ageing is well-known in fruit flies and worms (Kapahi et al. 2010).

### Other conclusions reported in the literature

#### Copper and iron ion transport

It has been shown that deficiencies in iron and copper levels are linked with ageing-related diseases like Atherosclerosis and Alzheimer’s disease (Brewer 2007). It is interesting that two papers using supervised machine learning algorithms have found that interactions with proteins involved in copper and iron ion transport are good predictors of ageing/longevity, as follows.

In Song et al. (2012), *atp7* was predicted as ageing-related in fruit flies. This gene is involved in copper ion transport in fruit flies (Norgate et al. 2006).

The gene *ftn-1* (related to iron ion transport) was predicted (with a probability of 0.95) as a longevity gene in Li et al. (2010a). It has been shown that the lack of *ftn-1* caused a reduced lifespan when worms are under iron stress (Kim et al. 2004).

#### Comparing findings from machine learning and functional enrichment analysis

One recent study investigated pathways overrepresented in pro- and anti-longevity genes using both traditional functional enrichment analysis and a machine-learning-based feature selection method Fernandes et al. (2016). Although the two methods work in different ways, some overlap between their results was observed. For example, both methods found terms related to insulin signaling or growth to be significantly associated with anti-longevity genes in mice; and terms related to autophagy were found to be significantly associated with pro-longevity genes in *C. elegans* by both methods. Therefore, it seems that both functional enrichment and machine learning algorithms identified the major, most significant pathways associated with longevity genes. However, there were also many pathways that were only identified by each of the methods (Fernandes et al. 2016). This raises the question of which method is more accurate, which at the moment is unclear. Our interpretation is that possibly both methods provide complementary information and should be used together.

#### Physiological biomarkers

Besides the ageing/longevity-related studies at the genomic/proteomic level, we have identified works that use physiological markers to study the ageing process. Next we discuss two works following such approach.

In Putin et al. (2016), authors claim that the analysis of relative feature importance within deep neural networks trained to predict chronological age on humans helped deduce the most important features that may shed light on the contribution of individual biological systems to the ageing process. The systems were ranked in the following order of decreasing importance, according to the selected features: metabolic, liver, renal system and respiratory function. The ranking was created by counting the number of biomarkers coming from the different systems.

In Nakamura and Miyao (2007), the authors investigate good predictors of chronological age of humans to develop an expression to calculate the biological age score of individuals. The authors used a population of 86 men, which were evaluated annually during six years. Their chronological age (the target variable) and the result of physiological exams (the predictive variables) were recorded and used in this study. Using Principal Components Analysis (PCA) and logistic regression, the authors identified the following five candidate biomarkers of ageing for constructing the score of biological age: systolic blood pressure, forced expiratory volume in 1 second divided by height squared, hematocrit blood level, albumin blood level, and blood urea nitrogen level.

#### Methylation biomarkers

Besides biomarkers from physiological exams, some works analyse biomarkers coming from methylation profiles of individuals.

In Horvath (2013), the author uses the Elastic Net regression algorithm to predict biological age using samples of several DNA methylation sites coming from several tissues. The author has concluded that the ageing-related changes in the methylome indicate a cumulative ageing-related effect on epigenetic maintenance systems. Interestingly, the author has also identified that mutations in the *p53* gene in cancer cells result in lower age-acceleration, which reinforces the connections with *p53* and ageing highlighted in the "Supervised machine learning findings support the link between ageing/longevity and specific types of DNA repair" section.

In Hannum et al. (2013), the authors use a regression method to predict the chronological age given biological markers describing the state of the methylome of 656 human individuals. The final model selected 71 markers to predict age. The authors interpreted the regression model and concluded that the selected methylation markers were consistent with the current knowledge of ageing research, that is, the methylated genes associated with the selected markers were known to be ageing related. The authors have validated their findings using an independent dataset, which strengthens their conclusions. Nearly all 71 methylation sites selected by the model lay within or are near genes with known functions in ageing-related conditions, including Alzheimer’s disease, cancer, tissue degradation, DNA damage, and oxidative stress.

In Weidner et al. (2014), the authors also train a regression model to predict the chronological age of several individuals based on their methylation profiles. In this work they observe that cells affected with diseases associated with severe telomere attrition are predicted to be abnormally aged by their regression model.

## Discussion and conclusions

### Overall biological knowledge extracted from the supervised machine learning models

We can conclude, based on our analysis of the literature using supervised machine learning applied to ageing research, that several already known biological facts were corroborated by supervised machine learning algorithms. Namely, it was shown that interactions with DNA repair genes and proteins are good predictors of ageing-relatedness (Horvath 2013; Hannum et al. 2013; Weidner et al. 2014), in special DNA repair proteins closely related to the Ku protein family (Freitas et al. 2011; Jiang and Ching 2011), the WRN protein (Jiang and Ching 2011; Li et al. 2010a; Fang et al. 2013) and the p53 protein (Fabris et al. 2016).

We have also observed that several works (Li et al. 2010a; Song et al. 2012; Feng et al. 2012; Li et al. 2010b) concluded that ageing-related proteins tend to be highly connected and seem to play a central role in molecular pathways. Additionally, works link ageing/longevity with autophagy and apoptosis  (Kerber et al. 2009; Li et al. 2010b; Feng et al. 2012; Wan and Freitas 2013; Wan et al. 2015; Fang et al. 2013; Fabris and Freitas 2016); nutrient receptor genes (Fabris et al. 2016; Wan et al. 2015; Wan and Freitas 2013); and copper and iron ion transport (Song et al. 2012; Li et al. 2010a).

At a higher-level perspective, several physiological biomarkers were found to be ageing related. In Putin et al. (2016), biomarkers in the following systems were identified: metabolic, liver, renal system and respiratory function. In Nakamura and Miyao (2007), the authors highlighted systolic blood pressure, forced expiratory volume in 1 second divided by height squared, hematocrit blood level, albumin blood level, and blood urea nitrogen level.

Some works have tested the predictions of the data mining algorithms using an independent testing set, with instances from a different species (Fortney et al. 2010; Hannum et al. 2013; Horvath 2013). Unfortunately, predictions of classification algorithms were only corroborated through wet-lab experiments in one paper (Li et al. 2010a). We believe that a stronger integration between machine learning and wet-lab experimentation would help to push the application of interpretable machine learning algorithms in ageing research. Experimental corroboration of *in silico* predictions is important to validate the conclusions of machine learning algorithms, thus demonstrating the power of this approach.

This type of independent algorithm validation using wet-lab experimentation and independent test sets is particularly important when applying supervised machine learning to ageing research because of biases and omissions in the training data. Given our imperfect understanding of biological systems, uncertainty is intrinsic to biological research. As such, for most studies, training datasets will have imperfect data with omissions and biases in the sense that some genes have been much more studied than others. In studies of ageing, these problems will be further compounded by the immature nature of the field. One recent study evaluated how using datasets with genes more or less studied than average may result in biases in large-scale analyses in ageing (Fernandes et al. 2016).

As such, in spite of recent advances in the genetics of ageing (de Magalhães and Tacutu 2016), it is likely that many ageing-related genes are still unknown depending on the species of choice. For worms, large-scale systematic screens have been performed for genes and drugs affecting longevity, while, in for example mice, our understanding of manipulations of ageing is likely to be much more incomplete. In addition, functional annotation of proteins is also far from uniform and many human proteins remain uncharacterised. Therefore, models built on significantly incomplete training sets will fail to find ageing-related genes that were unlike the training instances. Hence, the estimated predictive accuracy results based only on these biased datasets will likely be different from the predictive accuracy of the algorithms when applied to independent datasets.

### Strategies for supervised machine learning model interpretation

Regarding the strategies to interpret supervised machine learning models, we have identified 4 broad types in the papers we reviewed:

1. *Interpretation based on data pre-processing algorithms* To guide interpretability, these works use either the decisions of feature selection algorithms (Wan et al. 2015; Wan and Freitas 2013; Huang et al. 2012; Fernandes et al. 2016), or the relative importance of the original features when constructing new ones (Nakamura and Miyao 2007). Using these data preprocessing techniques has the advantage that they can select relevant features for predicting the target variable in general, potentially finding biological patterns that are independent of the choice of classification or regression algorithm to be used later. A disadvantage of this approach is that, if the relevant features are selected without considering the classification or regression algorithm to be applied later, the set of selected features may not lead to a good predictive performance of that algorithm.

2) *Interpretation based on classification/regression model analysis* These techniques explore the supervised machine learning models *per se* to reach meaningful conclusions about the underlying biological problem. The power of this approach mainly depends on two factors: first, the predictive performance of the supervised machine learning model; and second, the interpretability potential of the model.
  Some works interpret decision tree-based models (Fang et al. 2013; Freitas et al. 2011; Fabris et al. 2016; Fabris and Freitas 2016), which are easier to interpret but, in general, are not the “state of the art” regarding predictive performance. Some works interpret models which are commonly regarded as having more predictive power, like SVMs and Deep Neural Networks (Putin et al. 2016; Jiang and Ching 2011). However, the interpretation of these models is much harder, as they are more abstract. One clear advantage of interpreting supervised machine learning models is the preciseness of the type of biological conclusions that can be retrieved. For instance, decision tree models can inform the user of very precise rules, indicating which values of some variable(s) predict a specific value of the target variable. However, this approach has the disadvantage of depending on specific classification algorithms, which may have biases that can limit, or even mislead, the interpretation of the generated models. For instance, some algorithms do not deal well with discrete features. In this case, a highly predictive discrete feature could be ignored because of a limitation of a particular classification algorithm.
  We can also interpret the coefficients of regression models, or analyse the selected features used in the regression model, e.g. to identify the most relevant features to predict age (Hannum et al. 2013; Horvath 2013; Weidner et al. 2014; Kerber et al. 2009). Note that this approach may have the undesired side effect of overlooking variables that are correlated, and therefore, may not be all present in the final model. A clear advantage of interpreting the model’s coefficients is that they give a direct indication of which features are more relevant to the predictions of the model.

3) * Interpretation based on statistical analysis* The works of (Li et al. 2010a; Song et al. 2012; Feng et al. 2012; Li et al. 2010b) rely on statistical tests of significance to check if the values of the features have different distributions depending on the value of the target variable of the proteins. These interpretation approaches draw very broad conclusions about correlations between variables, rather than making precise predictions based on specific values of variables, like e.g. decision tree models. In addition, most works apply such tests of significance in a univariate fashion, considering one feature at a time, which ignores the issue of feature interaction. However, this approach enjoys the benefits of a sound statistical basis.

4) *Analysis of classifier predictions* The last type of interpretation approach we have observed [in (Li et al. 2010b; Feng et al. 2012; Song et al. 2012)] is the analysis of the supervised machine learning model’s output, without analysing the model *per se*. This approach is, arguably, the most straightforward from a machine learning perspective, but has the drawback of limiting the depth of the knowledge extracted from the models. Ideally, the biologist using the model should be able to understand *why* the model made a certain prediction (Freitas et al. 2010). This would both increase the confidence on the prediction and allow for new biological knowledge to be extract from the model.

### Characteristics of distinct feature types

We have observed that the works we reviewed have interpreted models with very distinct types of features, each with its own characteristics. Next, we list the main feature types that were interpreted in the reviewed papers.

1. *Organism-level features* The features found in Putin et al. (2016) and Nakamura and Miyao (2007) describe the results of high-level biomarkers such as the result of blood and urine tests, anthropometric measurements, cardiovascular and respiratory functions. These features have the advantages of being able to make predictions at the level of the individual, and being, in general, easily interpretable by clinicians. The main disadvantage of using this feature type is that it provides little knowledge about the mechanisms of ageing, since these features usually measure the effects of the underlying ageing mechanisms, rather than their causes.
  Another organism-level feature type is found in Hannum et al. (2013), Weidner et al. (2014), Horvath (2013), which use the methylation profiles of human donors as features; and in (Kerber et al. 2009), which uses gene expression profiles of human donors as features. Interestingly, these features have the opposite advantage and disadvantage than the physiological features: they are harder to interpret, as they measure very low-level alterations in an organism’s state; but give, at the same time, very specific information that may be used to understand the underlying mechanisms of ageing.

2) *Protein/gene interaction features* Features derived from protein and/or gene interactions were the most common feature type used in the reviewed works  (Freitas et al. 2011; Fang et al. 2013; Fabris et al. 2016; Fabris and Freitas 2016; Song et al. 2012; Feng et al. 2012; Li et al. 2010b; Li et al. 2010a; Huang et al. 2012; Fortney et al. 2010). Each feature has the value ‘1’ (the positive value) if there is an interaction between two proteins or genes, and has the value ‘0’ (the negative value) if there is no *known* interaction between genes or proteins. The positive value of this feature type encodes a precise relationship between two proteins or genes that is easy to understand and can help shed light into new ageing-related mechanisms.
  Interpreting the negative value of this feature type, however, is not so straightforward. A negative value for a feature means that there is *no evidence* for an interaction, not that the interaction does not exist. This must be taken into consideration when interpreting this feature type. Another disadvantage of using this feature type is that no interaction information is available for many genes or proteins of interest, hindering the use of this feature type.

3) *Functional annotation features* The works of Freitas et al. (2011), Wan and Freitas (2013), Wan et al. (2015), use features derived from ontologies that annotate proteins and genes using well-defined terms (like the Gene Ontology). The principle is that some annotations, which are not directly related to ageing, are good predictors of ageing-related processes. This approach is in agreement with the current knowledge about ageing, which has detected, for instance, that processes involved in nutrient recognition and growth are also linked to ageing modulation in several species (de Magalhães, 2011). Therefore, using this feature type can be useful for finding new biological processes that are also connected to ageing.
  A disadvantage of using such features is that, like the previously discussed interaction features, the negative value of a feature does not usually encode *absence* of function, just lack of evidence (so far) for that particularly function. Another issue is that an effective analysis of this feature type may require the adaptation of existing machine learning algorithms. This is the case because the hierarchical structure of gene ontologies results in many hierarchically redundant feature values, since a positive (negative) value for one feature imposes a positive (negative) value in all ancestors (descendants) of that feature in the hierarchy. Feature selection methods adapted to cope with this hierarchical feature redundancy are discussed in Wan and Freitas (2013); Wan et al. (2015).

### Concluding remarks

We conclude from the papers we reviewed that supervised machine learning algorithms are being applied in several ways to help biologists understand the biology of ageing. Not surprisingly, several already known biological facts were corroborated by supervised machine learning algorithms, and new ageing-related hypotheses have been put forth with the help of supervised machine learning algorithms and corroborated by wet lab experimentation or independent validation in other datasets.

Regarding the interpretation of supervised machine learning models, we have identified and discussed four broad approaches: (1) *interpretation based on data pre-processing algorithms*; where conclusions are not derived from the model *per se*, but from some pre-processing step. (2) *interpretation based on classification or regression model analysis*; where the biological conclusions are derived from the induced regression or classification model. (3) *interpretation based on statistical analysis*; where the biological conclusions are derived from statistical analysis, usually tests of statistical significance involving the feature values conditioned on the class of interest. (4) *analysis of classifier predictions*; where the prediction of the supervised machine learning models are analysed to extract biological insights.

We have also identified and analysed three types of features used in the works we have reviewed, these are: (1) *organism-level features*; which are features derived from measurements made in individuals of a population, instead of more generic species-level features. (2) *Protein or gene interaction features*; features that encode the information of whether a particular gene or protein interacts with other genes or proteins. (3) *Functional annotation features*; features that encode the information that a particular gene or protein is annotated with a label from a controlled vocabulary (such as the Gene Ontology) that represents a function performed by the gene or protein.

Finally, we argue that as the amount of ageing-related data increases, supervised machine learning tools have a bigger potential to help experts studying the biology of ageing. Arguably, this potential can only be fully realised if the predictions of supervised machine learning algorithms are properly validated in independent test sets or through wet-lab experimentation, which has been rarely done in the literature.
